# The process of slowing down in clinical reasoning during ultrasound consultations

**DOI:** 10.1111/medu.14365

**Published:** 2020-09-14

**Authors:** Marleen Groenier, Noor Christoph, Carmen Smeenk, Maaike D. Endedijk

**Affiliations:** ^1^ Technical Medical Centre Technical Medicine University of Twente Enschede The Netherlands; ^2^ Center of Evidence Based Education Faculty of Medicine Academic Medical Center University of Amsterdam Amsterdam The Netherlands; ^3^ Department of Educational Sciences Faculty of Behavioral, Management, and Social Sciences University of Twente Enschede The Netherlands

## Abstract

**Objectives:**

In clinical reasoning, clinicians need to switch between automatic and effortful reasoning to solve both routine and non‐routine problems. This requires the ability to recognise when a problem is non‐routine and adapt one's reasoning mode accordingly, that is to ‘slow down’ the reasoning process. In the current study, we explored the process of these transitions between automatic and effortful reasoning by radiologists who performed ultrasound examinations during consultations at the polyclinic.

**Methods:**

Manifestations of slowing down in clinical reasoning were explored in 41 out‐patient consultations performed by five radiologists. Interviews before and after the consultations were combined with observations during the consultations to obtain proactively planned triggers, slowing down manifestations and situationally responsive initiators. Transcripts of the interviews and field notes of the observations were coded. The constant comparative method was used to classify slowing down manifestations.

**Results:**

In thirteen of the 41 consultations, slowing down moments were observed. Four manifestations of slowing down were identified: shifting, checking, searching and focusing. These manifestations mainly differed in how long radiologists maintained effortful reasoning, varying from very short periods (shifting and checking) to sustained periods (searching and focusing). Unexpected patient statements and ambiguous ultrasound images initiated the slowing down moments.

**Discussion:**

The results from this study contribute to understanding how clinicians transition from automatic to effortful reasoning. Also, this study revealed two sources of initiators of this transition in radiologists’ consultations: statements made by the patient and conflicting or ambiguous visual information, in this case from ultrasound images. Natural variations in patient statements and visual information can be used as input of what might be meaningful variation in the domain of radiology education to support expertise development.

## Abstract 2

In this paper, Groenier et al. demonstrate how radiologists transition from near‐automatic to effortful reasoning to illustrate how clinicians 'slow down' the reasoning process.

## 1 INTRODUCTION

Clinical reasoning is at the heart of clinical expertise. According to dual‐process theory, doctors rely on two modes of reasoning when diagnosing a patient: a near‐automatic pattern detection mode and an effortful analytical mode.^1^ In the latter mode, biomedical knowledge might be addressed, additional information can be gathered, or the current problem is actively compared to past experiences in order to fuel problem‐solving, especially in non‐routine cases.^2^ Identifying whether a problem is routine or not, and adapting one's reasoning mode accordingly, is of key importance for accurate diagnosis.^3^

Rapid pattern detection processes are a quick and efficient way to arrive at a diagnosis but relevant attributes or alternatives might be overlooked.^4^ Careful and systematic exploration of all options is, however, time‐consuming and does not necessarily increase diagnostic accuracy.^5^ In some situations, expert clinicians might apply pre‐existing knowledge to non‐routine situations without the ability to see greater complexity.^4^ In practice, this means that a problem can appear as routine, while it is a non‐routine problem which might require a different, more analytical approach. In case of non‐routine situations, such as problems with conflicting, unexpected or ambiguous information, clinicians need to make a transition from automatic to deliberate, analytical reasoning.^6^

While dual‐process theory presents two cognitive modes, switching between them requires metacognitive monitoring.^7^ In line with the integrated model of clinical reasoning,^8^ metacognition—defined as the capacity to monitor and control one's thinking—may also take place on two different levels: an analytical, deliberate mode and a more experience‐based less conscious mode. Moulton et al^7^ explicitly open up the possibility that the process of slowing down can also be less consciously directed, but more research is needed to better understand these processes.

In line with Moulton et al,^9^ we argue that mere proficiency in operating in both modes is not enough to be an expert. This encompasses also the ability to identify when to slow down from the near‐automatic mode towards the effortful mode. Previous research in the domain of surgery has identified key characteristics of this transition, called ‘slowing down’.^7^ Research studying these processes in action outside the operating room is still lacking. Therefore, the aim of this study was to uncover the processes of slowing down in the radiology domain during patient consultation to inform the design of clinical reasoning training.

Moulton et al^7^ state that ‘slowing down when you should’ concerns metacognitive monitoring where an expert responds effectively in the moment and transitions appropriately from near‐automatic reasoning to analytical reasoning. They state that as clinicians face many uncertainties during their daily clinical activities, they need to be competent at detecting, understanding and responding effectively to environmental cues, and in other words, they need to maintain good situation awareness.^10^ Good situation awareness—an accurate and complete state of knowledge about the current situation—requires that a clinician has sufficient attentional capacity to attend to these critical cues from the environment. These critical cues from the environment can be triggers for recognising that application of the near‐automatic problem‐solving strategy does not deliver the desired or expected outcomes, that essential information might be lacking, that assumptions need to be scrutinised and that one should reconsider the problem‐solving strategy adopted.

Moulton et al^11^ presented a conceptual framework that focuses on the initiators of and factors influencing this transition. Initiators can be proactively planned, for example, constructing a problem representation prior to contact with a patient, or they can be situationally responsive, for example, when unexpected events occur. In a previous study, Moulton et al^9^ identified different forms of slowing down during surgery: stopping, removing distractions, focusing more intently and fine‐tuning. However, it is unclear to what extent these manifestations of slowing down are domain‐dependent or that they can be found in other domains than surgery.

### 1.1 Research objectives

Although many clinicians diagnose patients on a daily basis, to our knowledge no empirical research has focused on the process of slowing down during clinical reasoning. Acquiring extensive knowledge and skills in a domain is essential to becoming an expert. However, to flexibly apply the knowledge and skills when the situation demands it requires education and experiences that support learning to transition from near‐automatic to effortful reasoning and vice versa.^12^ Mylopoulos and Regehr^13^ state that current medical education results in training for pattern recognition, or near‐automatic reasoning, rather than mindful practice aimed at transitioning flexibly between modes of reasoning. Therefore, insights in the processes of slowing down can help educators to support learners how to apply and switch between rapid and effortful modes of clinical reasoning.^14^

In our study, we identify and describe the transitions from the routine to the more effortful mode of clinical reasoning by radiologists during a consultation at the polyclinic. We explore to what extent the conceptual framework of slowing down can be applied in a different clinical context. More specifically, our research question was: what are different manifestations of slowing down that we can observe during clinical reasoning and what initiates these manifestations of slowing down?

## 2 METHODS

### 2.1 Setting

The study was conducted at the Radiology department of the Amsterdam Medical Centre (AMC). We examined radiologists’ out‐patient consultations during which an ultrasound examination was performed over a period of one month. Ethical approval was obtained from the AMC and the Netherlands Association for Medical Education.

### 2.2 Participants

**FIGURE 1:**
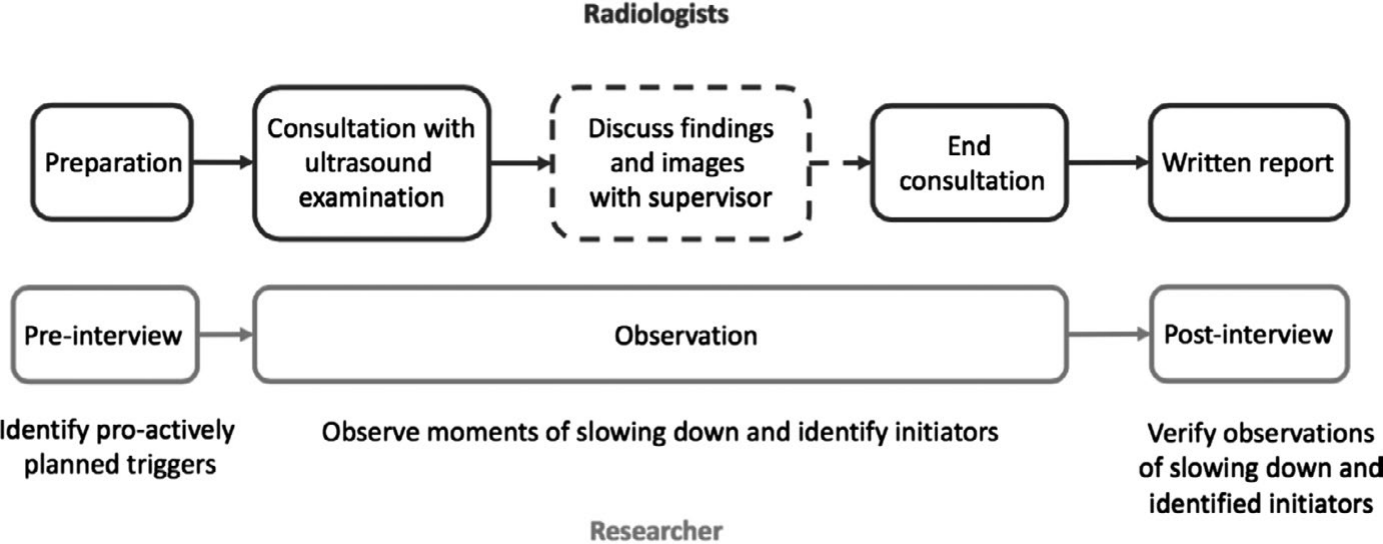
Overview of the study design. The top half represents the procedure for the radiologists, the lower half for the researcher (CS). The dotted lines represent the adjusted procedure for the residents

As slowing down manifestations could be related to expertise level, we purposefully sampled five radiologists with a broad range of expertise level, including four residents (experience range = 1.5‐5 years) and one attending (21 years of experience). All invited radiologists accepted our invitation (Figure 1).

All patients that were seen by the five radiologists were asked to participate in the study. Some patients did not give informed consent, for example, in case of underage patients. This resulted in a total of 41 observed out‐patient consultations included in the study, ranging from 5 to 12 consultations per participating radiologist.

### 2.3 Procedure

Radiologists were told that the aim of the research project was to identify characteristics of clinical reasoning. The process of slowing down and corresponding triggers was examined via triangulation of three different measures. First, a pre‐interview was held during participants’ preparation of a consultation to identify proactively planned triggers while creating a problem representation and differential diagnosis. Second, the consultation was observed by one of the researchers (CS) to identify slowing down moments and situationally responsive initiators. As the residents discussed each patient with their supervisor requiring them to verbalise their clinical reasoning, we also observed these discussions. Finally, in the post‐interview the slowing down moments and the initiators identified by the researcher were verified with the participant.

### 2.4 Interviews and observations

The pre‐ and post‐interviews were semi‐structured. The interview topics of the pre‐interview were as follows: (a) the type of patient problem, (b) what the participant expected to find, and (c) how he or she would approach the ultrasound examination. Proactively planned triggers that could lead to slowing down were discussed without informing the participants of the explicit goal of the study to avoid influencing them.

For the observations during the consultations, a similar approach was used as described by Moulton et al^9^. During the consultation, the researcher (CS) marked any verbal or non‐verbal cues indicating a possible slowing down moment. Examples of non‐verbal cues were as follows: (a) facial expressions of confusion, and (b) focusing more intensely, for example taking a closer look at the ultrasound image. Verbal cues were as follows: (a) timing and content of questions asked, (b) the amount of questions, (c) delayed or no verbal response to patient, and (d) expressing incomprehension.

The post‐interview was conducted with the radiologist and supervisor (if applicable) directly following the consultation to verify observed slowing down moments, identify missed slowing down moments and discuss possible triggers. The researcher started with open questions about marked verbal and non‐verbal cues to verify an observed slowing down moment, followed by more specific questions if necessary. Second, anticipated events and triggers mentioned in the pre‐interview were discussed. Third, events that did not happen as planned and deviations from the typical structure of a consultation were discussed as possible slowing down moments.

### 2.5 Data preparation and selection

All 41 pre‐interviews, consultations and post‐interviews were audiotaped, subsequently transcribed verbatim and coded in Atlas.ti. Separate codebooks were developed using a deductive approach, informed by Moulton et al’s theoretical framework of slowing down,^9^ combined with an inductive approach, by identifying various themes and concepts in the data. Based on the transcriptions and field notes, non‐verbal and verbal cues were identified that could indicate a manifestation of slowing down. Questions could indicate slowing down, for example when anamnesis questions were asked at the end of a physical examination. An increase in questions from the participant could indicate uncertainty about the differential diagnosis and the amount of questions is therefore used as an indicator of slowing down. These interpretations were verified in the post‐interview. Only those consultations in which slowing down was *observed* and *confirmed* by the participant were selected, resulting in 13 consultations (range = 1‐5 per participant).

### 2.6 Team reflexivity statement

The interviewer/observer (CS) was a communication and educational scientist in training, and she received training to observe medical practice. The interviewer took a ‘back seat’ approach during the consultations and did not interrupt them. Two researchers of the team (NC and ME) have a background in educational sciences with expertise on metacognition and professional learning. One of the researchers (MG) was a human factors researcher with a background in skill acquisition and trained as a mixed methods researcher. All researchers had a non‐clinical background.

### 2.7 Data coding and analysis

First, we determined for the 13 consultations the number of slowing down moments and what type of initiator (proactively planned or situationally responsive) was associated with a slowing down moment. See Appendix 1 for summaries of the codebooks. Second, slowing down moments were categorised following the constant comparative method set out by Glaser.^15^ The constant comparative method specifies that the slowing down moments are compared for similarities and differences to form categories and characteristics of these categories. Each new slowing down manifestation was compared to the already defined categories and their characteristics. Over time these characteristics became more integrated with each other. This process resulted in an emerging ‘property theory’,^15^ specifying the manifestations of slowing down and their associated characteristics. On several occasions during data analysis, the research team discussed the interpretation of slowing down moments and categorisations. At the end of the data analysis, a second coder who was not part of the research team examined the categorisations as an independent check.

## 3 RESULTS

Based on the analysis of the 13 consultations with slowing downs moments, we identified four distinct manifestations of slowing down and various initiators.

### 3.1 Shifting

Shifting (n = 2) occurred when a radiologist encountered unexpected clinical information that did not support any of the differential diagnoses but supported a (completely) different diagnosis that was not considered previously. During the ultrasound examination, anamnesis questions were asked that were not related to any of the diagnoses considered before, a shift in diagnosis was made, and the previous differential diagnoses were discarded. This manifestation of slowing down takes place within a short time span as the new diagnoses were quickly confirmed.

Shifting was initiated by new information provided by the patient or unexpected information from the ultrasound image. For example, during the ultrasound examination of an elderly woman, who was referred with complaints about her thumb joint, the radiologist discovered many small blood vessels on the ultrasound image. The radiologist reflected on this moment during the post‐interview:

I already told you [the researcher] that an abrasion in the thumb can be a consequence of eh, diseases, but lumps, such as these, that is quite strange. What surprised me was that I saw all those colours on the ultrasound, everywhere I looked […] I saw […], look, I saw those colours everywhere. Many blood vessels and much too small and those are inflammatory blood vessels, which can fit very well with rheumatism. A rheumatological disease. Two new hips for someone aged 60, that is actually a bit too young. Rad01

### 3.2 Checking

Another form of slowing down we found was Checking (n = 3): the radiologist consciously (double) checked additional clinical information that might support a particular diagnosis. This double checking was performed because the additional information indicated a less likely or rather profound diagnosis and the radiologist paused while double checking the information to be sure. Checking was recognised by verbal cues: multiple pauses during the consultation were observed as the radiologist verbally checked information with the patient about less likely differential diagnoses. Similar to Shifting, the transitions made during Checking have a short time span, but they consisted of multiple short transitions rather than just one.

Checking was initiated in our study by an unlikely ultrasound image. For example, in a consultation with a 50‐year‐old male patient with complaints about a worn shoulder, the ultrasound image indicated that the muscles were actually torn. The resident subsequently checked the patient history, intensity of the pain and the cause of the complaint to check this unlikely diagnosis. As this participant explains in a discussion with the supervisor:

Supervisor: ‘At the same time, this isn't really a frequent diagnosis, that the muscle is completely torn’.

Resident: ‘No, that is true. Therefore, I included a few things to check if this confirmed it. In this case it was of course […]. From the image it is actually immediately apparent, but then it is still a good thing to have some affirmative information’.

Rad02

### 3.3 Searching

In our study, Searching (n = 4) occurred because the radiologist was confronted with clinical information he or she could not explain. The radiologist then attempted to exclude differential diagnoses. Radiologists looked confused or verbally expressed confusion about the diagnosis under consideration. Additionally, anamnesis questions related to various differential diagnoses were asked during the ultrasound examination. In most cases, no final diagnosis was reached and patients were often referred for further examinations (eg a computed tomography scan). The radiologist made the transition to the effortful mode and gathered information to try and match this with his or her own prior knowledge. In most cases, deliberate reasoning lasted longer, often until the end of the consultation and no definite diagnosis was given. However, in a few cases the pieces of information fitted together in the end and a diagnosis was reached.

Searching was in our study always initiated by an ultrasound image that puzzled the radiologist. The ultrasound image showed information that the radiologist could not explain and therefore had to search for additional information, or the ultrasound image showed no abnormalities, while abnormalities were expected. In most of the cases, the radiologists drew a (preliminary) conclusion during the consultation, only to express doubt and consider other diagnoses or request additional examination to find support for a new differential diagnosis. For example, this radiologist shared his doubts about the diagnosis with the patient:

I see something black coming from that joint, a fluid with small white dots in it and that is something I do not understand. That is something I really have to think about, especially with what you [the patient] have been telling me. Rad03

### 3.4 Focusing

The last form of slowing down that was observed was Focusing (n = 3). The radiologist started working more focused while examining acquired clinical information to be certain he or she did not miss any vital information. Focusing only occurred during follow‐up consultations and could be characterised by a *lack of questions* during the ultrasound examination, looking more focused at the ultrasound screen in silence, pressing the probe harder or adjusting the procedure. Radiologists continued in a more focused state until the required clinical information was retrieved or until he or she was confident that something (eg a metastasis) was not present.

Focusing was initiated by a blurred ultrasound image (a technical issue) or because the image showed no abnormalities. This marked the transition to a more effortful mode, and the radiologist, for example, needed to press harder with the ultrasound probe or adjust the examination procedure:

Yes, then I really thoroughly inspect the segment whether I can see it. Also, as he [the patient] is now watching along with me, I look more focused: haven’t I missed something that I could not quite get on screen?’ Rad04

There was no indication that the four manifestations of slowing down were person specific. Combinations of manifestation of slowing down within a single consultation were not observed, but radiologists did show multiple forms of slowing down across consultations.

## 4 DISCUSSION

Clinical reasoning is an essential part of clinical expertise. Quick and accurate diagnostic reasoning depends on switching flexibly between two modes of reasoning when the situation demands it: near‐automatic pattern detection and deliberate, analytical reasoning.^9^, ^16^ The purpose of this study was to examine the transition between these two types of reasoning modes, also called moments of ‘slowing down’,^7^, ^9^, ^11^ in the clinical reasoning process.

We observed characteristics of the transition from more automatic to analytical reasoning, that is moments of slowing down, during radiologists’ out‐patient consultations. Four qualitatively different manifestations of slowing down, along with different initiators, were identified: *Shifting, Checking, Searching* and *Focusing*. Most slowing down moments were associated with unplanned events,^11^ which in our study consisted of statements made by the patient and the ultrasound images. Images could contain unexpected, unlikely or unknown information or the radiologist expected to see something but the information was not there. Patient statements were only related to *Shifting* and not to other manifestations of slowing down. No proactively planned initiators, that is factors that were identified prior to the consultation, were associated with slowing down moments in our study.

The observed slowing down moments were characterised by differences in the ‘investment of cognitive effort’ (p. 1575) as Moulton et al^9^ describe it. The radiologists in our study showed short periods of deliberate reasoning (as seen in Shifting and Checking) or more prolonged periods (Focusing and Searching). The radiologists detected cues from the environment signalling a possible non‐routine situation and reacted by slowing down their reasoning. This suggests that they successfully applied metacognitive skills in these situations to adapt to changing conditions.

Our findings partly overlap with the study on slowing down during surgery^9^ (see Table 1 for an overview). Focusing more intently during surgery corresponds to *Focusing* of radiologists and Fine‐tuning corresponds to *Checking*. For *Shifting* and *Searching*, no equivalent was found in the surgery context, indicating that this might be specific to the context of clinical reasoning.

**TABLE 1:** Manifestations of slowing down for surgery and clinical reasoning

Manifestation of slowing down	Domain	
	Surgery	Clinical reasoning
Stopping	The surgeon stops the operative procedure.	
Removing distractions	The surgeon removes distractions from the environment.	
Focusing (more intently)	The surgeon withdraws from extraneous conversation or distraction but proceeds without removing or controlling the environmental distractions.	The radiologist withdraws from conversation with the patient and works in silence for longer periods.
Fine‐tuning/ checking	The surgeon continues to engage in extraneous conversation or pauses momentarily to focus on the operative procedure.	The radiologist verbally checks information with the patient about a less likely differential diagnosis several times in a short time period.
Shifting		The radiologist asks anamnesis questions not related to any of the diagnoses considered before and a shift in diagnosis is made.
Searching		The radiologist looks confused or verbally expresses confusion and asks multiple questions related to several differential diagnoses.

These differences might be caused by contextual differences between specialties, such as the amount of interaction with the environment or time to respond to changes in the environment. Manifestations of slowing down in radiology might therefore be more similar to specialties such as dermatology, rehabilitation and otolaryngology than to the more action‐oriented and teamwork‐dependent specialties such as surgery or emergency medicine. Our study implies that manifestations of slowing down are, at least partly, context‐bound.

According to adaptive expertise theories, being able to recognise that a situation demands a different approach is related to a deep conceptual understanding of a domain.^17^, ^18^ This would explain the context‐bound nature of the manifestations of slowing down and their initiators. Understanding how expertise develops in a particular domain thus requires a broader perspective that not only takes knowledge and skill acquisition of the individual into account but also how an expert interacts with the environment.^19^, ^20^ Which cues will be detected from this interaction with the environment depends on the domain knowledge the clinician has.^7^, ^10^ The representation of a clinician's domain knowledge in that sense guides which cues will be picked up but, more importantly, how they are interpreted.^10^ In our study, we identified different manifestations of slowing down and identified different types of initiators that could have cued the radiologist to slow down. However, it was unclear whether they were ‘slowing down *when they should*’ and whether the identified initiators were sufficient to trigger these processes or whether there was also an influence of other contextual or personal characteristics that were beyond the scope of this study. There might have been instances that required the radiologist to slow down, but critical cues were missed, or situations in which the same cues were present, but the radiologist justly did not slow down. Further research is needed to understand how experts coordinate their attentional resources and interactions with the environment in clinical reasoning.

### 4.1 Implications for clinical reasoning instruction

In line with Marcum^8^ and Monteiro et al,^21^ we suggest that curricula explicitly address the relationship between pattern recognition and analytical processes in clinical reasoning. To improve the breadth and depth of experiential knowledge as the basis for both pattern recognition and analytical processes, there should be meaningful variation in the learning environment.^3^ Our study provides input for what might be meaningful variation in the domain of radiology education. Instruction for radiologists in clinical reasoning should take at least two sources of variation into account: (a) the information from the patient, that could either support or a diagnosis or make it less likely, and (b) the quality of the visual information acquired through the various visualisation modalities used in radiology.

### 4.2 Strengths and limitations

Our study successfully elaborated the findings from the Moulton et al^9^ study and, to our knowledge, we are the first to report observed moments of ‘slowing down when you should’ in the domain of clinical reasoning by radiologists. We examined slowing down across a variety of experience levels which resulted in a rich picture of slowing down manifestations. Including a group of residents allowed for a more direct observation of slowing down moments because they explicated their thoughts while discussing their cases with a supervisor.

Our study design had some limitations. First, our sample consisted of four residents and one expert, attending radiologist. Slowing down when you should is considered a hallmark feature of expertise. Our aim was to examine transitions from near‐automatic to effortful reasoning as part of expertise development; therefore, we included residents as well. Second, although we did not specifically brief the participants about the aim of our study, the actual aim of the study might be inferred from the post‐interview and could have influenced subsequent consultations of that participant. However, we did not see significantly different or more frequent slowing down behaviours in these subsequent consultations. Moreover, the aim of the study was not to make accurate observations of frequencies of processes of slowing down, but only on how processes of slowing down manifest during consultation. Therefore, we do not see this possible influence as a major threat to the validity of our findings. Third, the presence of the patient might have influenced the possibility to identify slowing down moments as some thoughts might not be expressed in front of a patient. Also, some patients did not give informed consent, for example because the patient was underage, but we have no reason to believe that this resulted in a biased selection of consultations. Finally, contrary to the Moulton et al^9^ study, we did not investigate the impact of slowing down on diagnostic accuracy. Therefore, we do not know whether slowing down did indeed result in less (severe) diagnostic errors.

### 4.3 Future directions

The current study was a first step into examining the phenomenon of ‘slowing down when you should’ in clinical reasoning of radiologists. Future studies might look at those moments where slowing down was adequate and when it was not. Large‐scale studies could answer questions about what combination of initiators under what circumstances with what type of clinicians (eg novice versus experts) trigger what type of slowing down behaviour. Also, the role of metacognitive processes in relation to ‘slowing down when you should’ is still unclear and needs to be investigated further. To what extent can self‐monitoring activities contribute to detecting, understanding and responding to cues from the environment or is slowing down a strictly bottom‐up, situationally responsive process?

## 5 CONCLUSIONS

Radiologists slowed down their practice in response to critical cues from the environment during out‐patient consultations at the polyclinic, suggesting they made transitions between automatic and effortful reasoning. Unexpected, ambiguous or conflicting information from patient statements and ultrasound images triggered these transitions. For future training of radiologists, we recommend to introduce clinical case studies from actual practice with characteristics of manifestations of slowing down and their initiators, such as the ones identified in our study, to practice clinical reasoning in a variety of contexts.

## CONFLICT OF INTEREST

None.

## AUTHOR CONTRIBUTIONS

MG contributed to the design of the work, analysis and interpretation of the data, drafting and revising the work and gave final approval for the version to be published. Also, MG agrees to be accountable for all aspects of the work. NC contributed to the design of the work, analysis and interpretation of the data, revising the work and gave final approval for the version to be published. Also, NC agrees to be accountable for all aspects of the work. CS contributed to the design of the work and conducted the study. Furthermore, CS contributed to analysis and interpretation of the data, drafting and revising the work and gave final approval for the version to be published. Also, CS agrees to be accountable for all aspects of the work. ME contributed to the design of the work, analysis and interpretation of the data, revising the work and gave final approval for the version to be published. Also, ME agrees to be accountable for all aspects of the work.

## ETHICAL APPROVAL

Ethical approval was obtained from the Amsterdam Medical Centre and the Netherlands Association for Medical Education.

## Appendix 1

### SUMMARIES OF THE CODEBOOKS FOR THE PRE‐INTERVIEW, OBSERVED CONSULTATION AND POST‐INTERVIEW

#### Pre‐interview codebook

**Table:**

Code	Definition
Request	
Complaint	Radiologist describes that the reason for the visit concerns a patient complaint.
Check	Radiologist describes that reason for the visit concerns a follow‐up appointment (eg to check for metastasis)
Differential diagnosis	
Hypothesis	Radiologist gives overview of illnesses that could be the cause of the patient complaint.
Judgement	In addition to the diagnosis the radiologist adds a judgement or a statement about what he or she would think of a particular diagnosis.
Initiators	
Patient specific—record	The radiologist describes what he or she already knows about the patient based on the patient record.
Patient specific—person	Radiologist explains what or what he or she will ask the patient. He or she explains how he/she will approach the anamnesis.
Procedure specific	Radiologist explains what he or she will do regarding the ultrasound examination.

#### Observed consultation codebook

**Table:**

Code	Definition
Acquaintance and contact	
	The patient and radiologist greet each other; the radiologist checks patient's demographic information.
Anamnesis	
Reason	The radiologist and patient discuss the reason for the visit, such as complaints about pain.
History	The radiologist and patient discuss the medical history of the patient such as previous illnesses, treatments and past experiences with medical interventions.
Medication	The radiologist or the patient mentions the use of medication.
Other clinicians	The radiologist or the patient mentions other clinicians that the patient is seeing.
Nature of complaint	The radiologist and patient discuss location, frequency and extent of pain or feeling of discomfort.
Intensity of complaint	The radiologist and patient discuss the intensity, severity or impact of the complaint.
Chronology of complaint	The radiologist and patient discuss the time frame such as onset and continuity of the complaint.
Cause	The radiologist and patient discuss the cause of the complaint.
Coherence	The radiologist and patient discuss social and situational aspects of the patient's complaint, such as accompanying symptoms, aggravations or relation with daily activities, as well as family history.
Conception and perception	The radiologist and patient discuss the patient's own ideas, experiences and expectations concerning the primary complaint.
Other	The radiologist and patient discuss other things, such as small talk.
Physical examination	
Description	The radiologist describes what he/she does/sees/feels or the patient asks about what the radiologist does/sees/feels.
Explanation	The radiologist explains what his/her interpretation of the visual data or physical examination is or the patient asks about this.
Instructions	The radiologist gives the patient instructions for the physical examination or the patient asks something about the instructions.
Other	The radiologist and patient discuss other things.
Diagnosis	
Hypothesis	The radiologist makes statements about possible diagnoses or doubts about the cause of the illness.
Final diagnosis	The radiologist draws his/her diagnostic conclusion or excludes a diagnosis.
Other	The radiologist and patient discuss other things.
Intervention	
Additional examinations	The radiologist or patient tells/asks something about additional (prior) examinations.
Treatment	The radiologist tells or the patient asks something about treatment.
Other	The radiologist and patient discuss other things.
Conclusion	
	The radiologist discusses the follow‐up, says goodbye and makes a record of the consultation.

#### Post‐interview codebook

**Table:**

Code	Definition
Request	
Complaint	Radiologist describes that the reason for the visit concerns a patient complaint.
Check	Radiologist describes that reason for the visit concerns a follow‐up appointment (eg to check for metastasis).
Differential diagnosis	
Hypothesis	The radiologist describes which different illnesses he or she considers likely based on what he or she has observed during the consultation.
Final diagnosis	The radiologist draws a diagnostic conclusion, classifying and explaining the patient's symptoms.
Initiators	
Patient specific—record	The radiologist describes what he or she already knows about the patient based on the patient record.
Patient specific—person	The radiologist refers to statements made by the patient made during the consultation, that is information gathered during the consultation.
Procedure specific	The radiologist explains the ultrasound images that were made and what can be seen on them.
Slowing down moments	
Positive	The radiologists states that he or she had to ‘slow down’, for example in terms of working more concentrated.
Negative	The radiologist states he or she did not notice ‘slowing down’, for example there was no need to work more concentrated.
